# Lanostane-Type Saponins from *Vitaliana primuliflora*

**DOI:** 10.3390/molecules24081606

**Published:** 2019-04-23

**Authors:** Maciej Włodarczyk, Antoni Szumny, Michał Gleńsk

**Affiliations:** 1 Department of Pharmacognosy and Herbal Medicines, Faculty of Pharmacy with Division of Laboratory Diagnostics, Wroclaw Medical University, Borowska 211a, 50-556 Wroclaw, Poland; michal.glensk@umed.wroc.pl; 2 Department of Chemistry, Faculty of Food Science, Wrocław University of Environmental and Life Sciences, Norwida 25, 50-375 Wroclaw, Poland; antoni.szumny@upwr.edu.pl

**Keywords:** *Androsace*, *Douglasia*, *Primula*, *Vitaliana*, Primulaceae, primrose, triterpenoid saponin, steroid saponins, lanostane, UHPLC screening

## Abstract

The phytochemistry of the genera *Androsace*, *Cortusa*, *Soldanella*, and *Vitaliana*, belonging to the Primulaceae family is not well studied so far. Hence, in this paper, we present the results of UHPLC-MS/MS analysis of several primrose family members as well as isolation and structure determination of two new saponins from *Vitaliana primuliflora* subsp. *praetutiana*. These two *nor*-triterpenoid saponins were characterized as (23*S*)-17α,23-epoxy-29-hydroxy-3β-[(*O*-β-d-glucopyranosyl-(1→2)-*O*-α-l-rhamnopyranosyl-(1→2)-*O*-β-d-glucopyranosyl-(1→2)-*O*-α-l-arabinopyranosyl-(1→6)-β-d-glucopyranosyl)oxy]-27-*nor*-lanost-8-en-25-one and (23*S*)-17α,23-epoxy-29-hydroxy-3β-[(*O*-α-l-rhamnopyranosyl-(1→2)-*O*-β-d-glucopyranosyl-(1→2)-*O*-α-l-arabinopyranosyl-(1→6)-β-d-glucopyranosyl)oxy]-27-*nor*-lanost-8-en-25-one, respectively. Their structures were determined by high resolution mass spectrometry (HRMS), tandem mass spectrometry (MS/MS), one- and two-dimensional nuclear magnetic resonance spectroscopy (1D-, and 2D-NMR) analyses. So far, the 27-*nor*-lanostane monodesmosides were rarely found in dicotyledon plants. Therefore their presence in *Vitaliana* and also in *Androsace* species belonging to the *Aretia* section is unique and reported here for the first time. Additionally, eleven other saponins were determined by HRMS and MS/MS spectra. The isolated lanostane saponins can be considered as chemotaxonomic markers of the family Primulaceae.

## 1. Introduction

Some plants are rich in secondary metabolites of a specific class, e.g., saponins. They can reach up to 10% of dry mass and, thus, are attractive for industrial usage. Among them, steroidal saponins are particularly abundant in monocotyledons, while triterpenoid saponins are abundant in eudicotyledons, with several exceptions. Among a few cases of medicinally valuable steroidal glycosides present in angiosperms, cardiac glycosides and their open-lactone analogs should be mentioned [1]. Other economically important compounds are appetite suppressants from the South African *Hoodia* sp., Euphorbiaceae [2] or male hormone imitators from *Tribulus* sp., Zygophyllaceae [3]. On the other hand, typical triterpenoids (C_30_) are rare in monocotyledons [4].

Taking under consideration the nomenclatural ambiguity of saponins, some researchers classify tetracyclic triterpenoids, for example, ginseng dammaranes, as steroids, and this term regularly appears in some papers [5]. With lanostanes: Some classify them as steroids because of the biosynthesis step of squalene cyclization and mutual conformation of the resulting rings [6,7,8], while others catalog them as triterpenoids by the presence of two methyl substituents in position 4 and count the total carbon number of this aglycone as 30 [7,9].

Lanostane homologs are uncommon in dicotyledonous plants [7]. They can be found in many Asparagaceae members like in ornamental muscari or squills [10] and conifers [11]. A variety of sea cucumbers should be mentioned as a non-vegetable lanostane source [12,13]. A considerable number of bioactive, non-glycosidic lanostanes was reported in some fungi, including the famous *Ganoderma* sp. [14,15].

Up to now, the Primulaceae family was known to be a source of triterpenoid saponins of the oleanane type [16] and several cucurbitacins [17], beside several unique flavonoids [18,19].

Performing the phytochemical screening and characterization of this family, we have developed rapid, useful, and universal UHPLC-MS and -MS/MS methods for saponin determination. Moreover, we described the isolation and identification of two dominant saponins from *Vitaliana primuliflora* Bertol., that were previously observed on thin layer chromatography (TLC) only [20]. Finally, we proved the occurrence of these two 27-*nor*-lanostane saponins in some species belonging to *Androsace* (in *Aretia* and *Douglasia* sections only). It is also the first communication describing plant *nor*-lanostanes outside the Asparagaceae family.

## 2. Results

**Figure 1:**
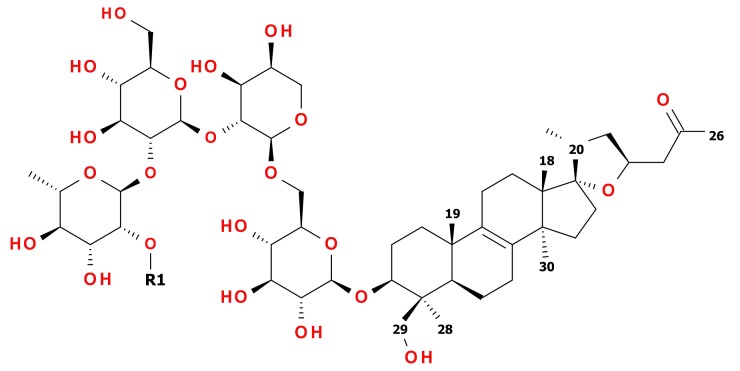
Structures of **12** (R1 = 1-β-D-Glc*p*) and **13** (R1 = H).

Hydroalcoholic plant extracts were prepared routinely and analyzed by UHPLC-MS and UHPLC-MS/MS in the negative mode as a part of a more comprehensive screening. The first examination of MS chromatograms revealed two main unidentifiable ions of high intensity, especially in *Vitaliana* species extracts. Later on, about 13 g of underground parts of *V. primuliflora* subsp. *praetutiana* were taken for extraction. Subsequently, with the help of semi-preparative flash chromatography on silica and HPLC on reversed phase, we have obtained approximately 40 mg of **12** and 36 mg of **13**, as amorphic white solids (almost 0.3% of starting dry mass each; Figure 1). Both compounds could form a stable foam at a concentration of 0.1 mg/mL. The isolation was performed in mild conditions to avoid any artifact formation. All fractionation steps were monitored by TLC/HPLC.

### Structure Elucidation of New Saponins

The HRMS spectrum revealed the molecular formula of **12**, the most abundant peak (R_t_ = 13.98 min, UV_max_ = 199 nm) to be C_58_H_94_O_27_ ([M − H]^−^ = 1221.5910 *m*/*z* (calcd.) vs. 1221.5889 *m*/*z* (meas.), err. 1.7 ppm). The MS/MS fragmentation indicated the gradual loss of hexose, deoxyhexose, hexose, pentose, and hexose. The lack of coexisting significant fragmentation peaks suggested that the sugars were linearly arranged in one glycone chain. The resulting aglycone was found to have a formula of C_29_H_26_O_4_ ([M − H]^−^ = 457.3323 *m*/*z* (calcd.) vs. 457.3310 *m*/*z* (meas.), err. 2.9 ppm). Sugar identity (glucose, arabinose, rhamnose) was confirmed after acidic hydrolysis on TLC as described previously [21].

**Figure 2:**
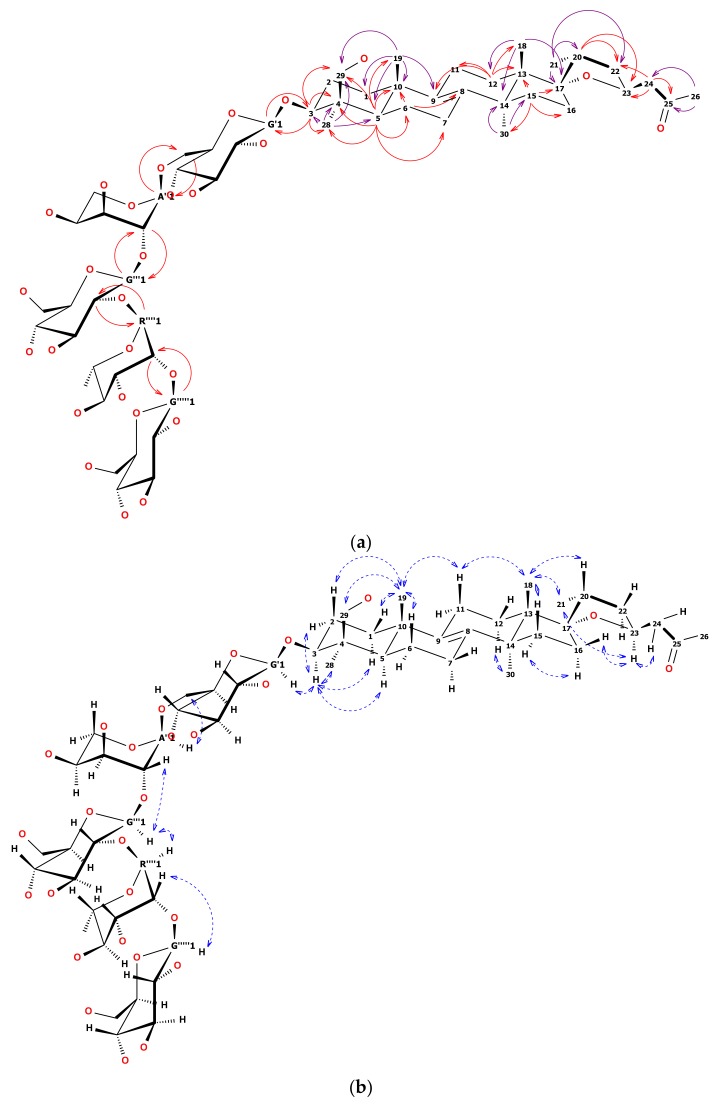
(**a**) Structure of **12**. Solid, one-way arrows represent the key HMBC correlations (violet from methyl groups, red from other hydrogens). (**b**) Structure of **12**. Dashed, blue, two-way arrows represent the key NOESY correlations

^1^H-NMR and heteronuclear single quantum coherence (HSQC) spectrum of **12** showed five anomeric signals. Two of them, observed as narrow doublets, were initially assigned to α-L sugars while three wide doublets were key to β-D sugars [22]. Overlapping ^1^H signals were resolved by the examination of HSQC and heteronuclear multiple bond correlation (HMBC) together with total correlated spectroscopy (TOCSY) and finally defined by heteronuclear two bond correlation (H2BC). Sugar chain linearity and positions of substitution were confirmed by 2D-NMR as shown in Figure 2a,b. Briefly, anomeric hydrogen of first glucose moiety was HMBC correlated with C3 of the aglycone and HMBC correlation from H3 to C1 of the first glucose was observed. This pointed out the place of substitution of the aglycone with the sugar chain. The C6 signal of the first Glc*p* was shifted downfield by 6 ppm relative to the non-substituted Glc*p*. This chemical shift difference suggested Ara*p* was linked to the C6 of the first Glc*p*. Anomeric hydrogen of the second glucose unit was correlated not only with C2 of arabinose but also with C1 of arabinose. That was the reason the second glucose was linked (1→2) to arabinose. C2 signal of the second glucose was shifted downfield relative to the non-substituted Glc*p*. That remarked that this sugar was substituted with the next one (rhamnose). HMBC correlations verified that supposition. The observation of highly shifted C2 of Rha*p* (up to 83 ppm) together with long-range HMBC of an anomeric proton from the third Glc*p* determined the position of substitution at the end of the sugar chain. The ^13^C chemical shifts of the glycone part of **12** were strictly similar to those reported in the literature [23,24,25].

The general pattern of the ^13^C-NMR spectrum showed similarity of the aglycone of **12** to the well-known eucosterol [25]. Seven methyl groups (two of them split by nodal hydrogen), together with 14 methylene groups and eight quaternary carbons were observed. One of the methyl doublets was ascribed to the sugar unit (Rha*p*) while the second was ascribed to C21 of eucosterol. The examination of long-range HMBC correlations of the rest of the methyls and of well-separated H3 and H5, let us build a skeleton of the lanosterol-like molecule. Among quaternary carbon signals, the highest shift at 208.6 ppm was assigned to the carbonyl. The two double-bond forming carbons were observed at about 135 ppm (close to pyridine-*d5* signals). Spiro carbon was visible at 96.2 ppm. The remaining four quaternary carbons were fixed to aglycone nodes, each substituted with the methyl group. Four methylene groups were assigned to terminal carbons of sugars (3× glucose, 1× arabinose). Another one was assigned to oxygenated methyl (C29, –CH_2_OH) in the close neighborhood of C5 and methyl C28 (based on HMBC and nuclear Overhauser effect spectroscopy (NOESY)).

Compared to eucosterol, C15 of **12** remained unsubstituted (based on TOCSY and HMBC correlations), while the side chain (C24–C26) seemed to be modified. It was observed, that the side chain showed a significant downfield shift of the terminal CH_3_ group from 7 to 30 ppm compared to reference [25]. Quaternary carbonyl was the nearest neighbor to this CH_3_ group and two CH_2_ hydrogens were HMBC correlated both with carbonyl and epoxy ring hydrogens and carbons. Thus the order of =CH_2_ and =CO in the C24–C26 chain had to be reversed compared to eucosterol. Detailed HMBC, TOCSY, and NOESY examination revealed the rest of well-separated signals typical for eucosterol (Table 1 and Table 2). All key correlations are visualized in Figure 2a,b.

We based the stereochemistry of the epoxy ring of **12** on an earlier report [4]. The ^13^C shifts of C16 and C17 together with strong NOE correlation between CH_3_18 and CH_3_21 methyl groups indicated the 17*S* and 20*R* conformation [26]. To prove it, we found that the H11*_ax_* at 1.95 ppm was NOE correlated with both methyl groups CH_3_18 at 0.90 and CH_3_19 at 0.94 ppm. Besides, CH_3_18 was NOE correlated with both H20 at about 2.02 ppm and CH_3_21, that suggested the β position of C20 in relation to the skeleton (resulting in 17*S* conformation). Secondly, a well-separated signal of H23 at 4.61 ppm was simultaneously NOE correlated with the methyl group CH_3_21 at 1.02 ppm and H16*_eq_* at 1.74 ppm. Moreover, none of the two H24 hydrogens was NOE correlated with the main skeleton hydrogens. Thus both H23 and CH_3_21 were assigned as α in relation to the skeleton (resulting in 20*R* and 23*S* conformation).

The neutral molecular formula of **13**, noticed as the second most prominent peak in MS chromatogram of *V. primuliflora* hydroalcoholic extract (R_t_ = 14.69 min, UV_max_ = 199 nm) was revealed to be C_52_H_84_O_22_ ([M − H]^−^ = 1059.5381 *m*/*z* (calcd.) vs. 1059.5359 *m*/*z* (meas.), err. 2.1 ppm). The MS/MS fragmentation of **13** was analogous to that of **12** (close pattern of fragments together with their relative intensities). However, there was a lack of terminal hexose. The resulting aglycone of **13** was found to have the same formula as **12**, C_29_H_26_O_4_ ([M − H]^−^ = 457.3323 *m*/*z* (calcd.) vs. 457.3331 *m*/*z* (meas.), err. 1.7 ppm). Sugar chain order was similar as in the case of **12** but with the third Glc*p* lacking and unsubstituted Rha*p* C2 (regular shift at about 72 ppm). Detailed examination of 1D- and 2D-NMR spectrum of **12** and **13** aglycones confirmed their identity.

Based on the abovementioned deduction **12** is (23*S*)-17α,23-epoxy-29-hydroxy-3β-[(*O*-β-d-glucopyranosyl-(1→2)-*O*-α-l-rhamnopyranosyl-(1→2)-*O*-β-d-glucopyranosyl-(1→2)-*O*-α-l-arabinopyranosyl-(1→6)-β-d-glucopyranosyl)oxy]-27-*nor*-lanost-8-en-25-one and **13** is (23*S*)-17α,23-epoxy-29-hydroxy-3β-[(*O*-α-l-rhamnopyranosyl-(1→2)-*O*-β-d-glucopyranosyl-(1→2)-*O*-α-l-arabinopyranosyl-(1→6)-β-d-glucopyranosyl)oxy]-27-*nor*-lanost-8-en-25-one. The structures of **12** and **13** are presented in Figure 1, while their chemical shifts are listed in Table 1 and Table 2. More HRMS fragmentation data together with retention times for compounds **12** and **13** with other observed and tentatively described triterpenoid glycosides **1**–**11** are arranged in Table A2.

## 3. Discussion

Taking under consideration all the Primulaceae species listed in Table A1, the newly described triterpenoid saponins **12** and **13** were detected by UHPLC-MS and UHPLC-MS/MS in the underground parts of the genus *Androsace*, in sections *Aretia* (five of six samples) and *Douglasia* (one of one samples) as well as in the cognate genus *Vitaliana* (four of four samples). The 27-*nor*-lanostane glycosides **12** and **13** were dominant among saponins detected by ESI-MS in the negative mode in all *Vitaliana* plants. The richest sample was *V. primuliflora* subsp. *praetutiana* (VPPR_B_2015), from which these two compounds were isolated (VPPR_B_2017). In samples derived from *Androsace cylindrica* and *A. obtusifolia*, the intensity of **12** was on a similar level as in *V. primuliflora* (VPRI_B_2015). In the remaining samples containing **12**, the concentration of this compound was lower than that in *V. primuliflora* subsp. *assoana* (VPAS_TK_2015). MS signal intensity of **12** was significantly higher than that of **13** in all *Vitaliana* plants and in *Androsace calderiana*, *A. lactiflora*, *A. obtusifolia*, *A. mathildae*, and *A. montana* (= *Douglasia montana*). In most samples containing **12**, it was also the best detectable saponin on an MS chromatogram (except *A. montana*, *A. mathildae*, and *A. obtusifolia*). *Androsace lehmannii*, the only examined *Aretia* member originating from Asia was transferred to section *Aretia* based on its morphological features [27]. We did not prove any of the newly discovered saponins in the analyzed sample of *A. lehmannii*. It may indicate that this species is chemically different from the rest of the *Aretia* members, and thus should be more precisely analyzed, also by botanists.

This is the first report on the occurrence of plant *nor*-lanostane saponins outside the Asparagaceae family. The new compounds were detected in samples originating from different locations, that makes our observations more reliable. All isolation procedures were conducted in mild and acid-free conditions. Nonetheless, we cannot exclude the possibility of the endophytic origin of isolated 27-*nor*-lanostanoids, as such observations were already published [28].

The assumption of extensive studies presented in this paper in part only was to develop an integrated model to screen many samples for the detection of a specific group of secondary metabolites, namely saponins. In our opinion, a detailed analysis of a broad range of related organisms may lead to observation and notification of some statistically significant rules. Usually, the problem is that only some of the plants in a selected botanical group are treated as important for public opinion. The reasons are enormous biomass gain or well-established pharmaceutical or medical properties. Our study shows that less popular plants could also be an interesting source of natural compounds.

Additionally, we would like to point out that amateur sourcing, breeding, or widespread cultivation of ornamental and exceptional plants (e.g., so-called ‘alpines’) may be a good measure in order to collect phytochemically interesting plants.

## 4. Materials and Methods

### 4.1. Plant Material

The plants were obtained from commercial suppliers, mainly from reputable nurseries. A precise list of seedlings suppliers and sample acronyms is available in Appendix A, Table A1.

For screening purposes, three seedlings each of: *V. primuliflora* Bertol., *V. primuliflora* subsp. *assoana* M. Laínz, *V. primuliflora* subsp. *praetutiana* (Buser ex Sünd.) I. K. Ferguson were used. Plants were documented by the author (M.W.). Vouchers (VPRI_B_2015, VPAS_TK_2015, VPPR_B_2015) were stored in a herbarium of the Department of Pharmacognosy and Herbal Medicines, Wroclaw Medical University. The plants were separated from the soil, carefully cleaned with tap water, separated into the underground and aboveground parts, allowed to dry in the shade for two weeks, and then stored in paper bags. Parts of plants were separately powdered right before further processing (Basic A11; IKA, Staufen, Germany), sieved (0.355 mm), and stored in darkness in airtight containers. *Androsace*, *Soldanella*, and other used plant species were processed in the same way.

Bergenia Nursery (Kokotów 574, 32-002 Węgrzce Wielkie, Poland; 50°01’21.9”N 20°06’03.0”E) was the supplier of a larger number of seedlings for the isolation of new saponins (VPPR_B_2017).

### 4.2. Chemicals

LC-MS grade water and formic acid were purchased from Merck (Darmstadt, Germany) while acetonitrile was obtained from Honeywell (Morris Plains, NJ, USA). Analytical grade chloroform was sourced from Chempur (Piekary Śląskie, Poland) and methanol from POCh (Lublin, Poland).

### 4.3. Instrumental Equipment

The Thermo Scientific UHPLC Ultimate 3000 apparatus (Thermo Fisher Scientific, Waltham, MA, USA) comprising an LPG-3400RS quaternary pump with a vacuum degasser, a WPS-3000RS autosampler, and a TCC-3000SD column oven connected with an ESI-qTOF Compact (Bruker Daltonics, Bremen, Germany) HRMS detector was used. The separation was achieved on a Kinetex RP-18 column (150 × 2.1 mm × 2.6 μm; Phenomenex, Torrance, CA, USA).

The manual Knauer 64 isocratic pump (Knauer, Berlin, Germany) was used for solid phase extraction (SPE; RP-18 cartridge of 10 g; Merck), flash chromatography (FC; 85 g of Si_60_ in a packed column of diameter 20 mm; Merck) and semi-preparative HPLC (100-5-C18, 250 × 10 mm × 5 μm; Kromasil, Bohus, Sweden).

^1^H-, ^13^C-, and 2D-NMR spectra were obtained on a Bruker Avance 600 MHz NMR spectrometer (Bruker BioSpin, Rheinstetten, Germany), operating at 600 MHz and 125 MHz respectively at 300 K, using standard pulse programs, and pyridine-*d5* (Armar AG, Döttingen, Switzerland) was used as the solvent. An internal solvent signal was used as a reference. NMR results are presented in Table 1 and Table 2. NMR data are presented in the Supplementary Materials (Figures S2 and S3).

### 4.4. Analytical Samples Preparation

Samples for UHPLC analyses were extracted (100 mg sample per 2 mL of 70% methanol) in an ultrasonic bath (15 min, 25 °C, 50% of power; Bandelin, Berlin, Germany). Then, 1 mL of each extract was filtered through a 0.22 μm PTFE syringe filter (Merck-Millipore, Darmstadt, Germany), diluted 100 times with 50% acetonitrile in water (LC-MS class) and stored at 4 °C before the analysis.

### 4.5. UHPLC-MS and UHPLC-MS/MS Analysis

The UHPLC-MS instrument was operated in negative mode. The HRMS detector was calibrated in the dead time of every single run with the Tunemix^TM^ mixture (Bruker Daltonics) with *m*/*z* standard deviation below 0.5 ppm. The analysis of the obtained mass spectra was carried out using Data Analysis 4.2 software (Bruker Daltonics). The key instrument parameters were: Scan range 50–2200 *m*/*z*, low mass set at 200 *m*/*z*, nebulizer pressure 1.5 bar, dry gas N_2_ with flow 7.0 L/min, temperature 200 °C, capillary voltage 2.2 kV, ion energy 5 eV, collision energy 10 eV and 30 eV (in separate runs). MS^2^ analyses were performed for ions in the range 400–1900 *m*/*z*, with collision energy gradient 40→170 eV.

The gradient elution system consisted of 0.1% formic acid in water (mobile phase A) and 0.1% formic acid in acetonitrile (mobile phase B). At the flow rate of 0.3 mL/min, the following elution program was used: 0→1 min (2→30% B), 1→21 min (30→50% B), 21→21.5 min (50→100% B), 21.5→25.5 min (100% B). The column was equilibrated for 5 min before the next analysis. Blanks were run after each sample to avoid any cross-contamination. Other parameters were: Column oven temperature 30 °C, injection volume 5 μL. The MS/MS fragmentations of **12** and **13** are presented in Supplementary Materials (Figure S1). LC-MS results are presented in Appendix A, in Table A2.

### 4.6. Isolation of Compounds ***12*** and ***13***

Based on UHPLC-MS results, VPPR_B_2017 underground parts were selected for semi-preparative purposes. The total amount of 12.875 g of underground parts was macerated three times with 70% methanol in water in the ratio 1:10. Combined extracts were diluted with water and applied to SPE. The fraction eluted with 70% to 85% methanol was concentrated to dryness in vacuo in 40 °C (Rotavapor V-100; Büchi, Flavil, Swiss), giving 346 mg (2.7% of initial dry mass). The first separation was performed by FC on 85 g of Si_60_ (Merck, Darmstadt, Germany), with chloroform→methanol gradient at flow rate 5 mL/min. Further, selected fractions were purified by semi-preparative HPLC on RP-18 in 75% methanol (isocratically) with a flow of 3 mL/min. As a result, 36.1 mg of **13** and 40.8 mg of **12** were obtained, corresponding to 0.28% and 0.32% of starting dry mass. To assure, that no artifacts were collected, **12** and **13** were confronted with primary extract by UHPLC-MS (conditions as described in Section 4.5).

## Figures and Tables

**Table 1:** ^1^H-(600 MHz) and ^13^C-(125 MHz) NMR chemical shifts of compound **12** (in pyridine-*d5*).

Position	δ_C_ (ppm)	δ_H_ (ppm)	δ_H_ (ppm)
C1	36.24 (t)	1.68 (m, 1H) *eq*	1.17 (td, *J* = 13.6, 3.5 Hz, 1H) *ax*
C2	27.97 (t)	2.27 (m, 1H) *eq*	2.00 (m, 1H) *ax*
C3	89.42 (d)	3.57 (dd, *J* = 11.8, 4.6 Hz, 1H) *ax*	
C4	44.91 (s)	-	
C5	52.29 (d)	1.28 (dd, *J* = 12.8, 4.6 Hz, 1H) *ax*	
C6	19.23 (t)	1.83 (dd, *J* = 13.2, 6.6 Hz, 1H) *eq*	1.51 (m, 1H) *ax*
C7	27.39 (t)	2.02 (m, 2H)	
C8	135.78 (s)	-	
C9	135.11 (s)	-	
C10	37.28 (s)	-	
C11	21.54 (t)	2.10 (m, 1H) *eq*	1.94 (m, 1H) *ax*
C12	25.76 (t)	2.35 (dt, *J* = 13.9, 9.0 Hz, 1H) *ax*	1.41 (m, 1H) *eq*
C13	49.32 (s)	-	
C14	51.30 (s)	-	
C15	32.65 (t)	1.67 (m, 1H) *ax*	1.38 (m, 1H) *eq*
C16	42.26 (t)	1.99 (m, 1H) *ax*	1.74 (m, 1H) *eq*
C17	96.22 (s)	-	
C18	19.82 (q)	0.90 (s, 3H) *ax* (β)	
C19	20.01 (q)	0.94 (s, 3H) *ax* (β)	
C20	44.47 (d)	2.01 (m, 1H) *ax*	
C21	17.97 (q)	1.02 (d, *J* = 6.8 Hz, 3H) *eq*	
C22	40.99 (t)	1.69 (m, 2H)	
C23	74.11 (d)	4.62 (m, 1H) *ax*	
C24	53.30 (t)	2.85 (dd, *J* = 15.3, 7.8 Hz, 1H) A	2.61 (dd, *J* = 15.3, 5.3 Hz, 1H) B
C25	207.59 (s)	-	
C26	30.83 (q)	2.21 (s, 3H)	
*nor*-C27	-	-	
C28	23.64 (q)	1.56 (s, 3H) *eq*	
C29	63.65 (t)	4.44 (m, 1H) A	3.66 (m, 1H) B
C30	26.84 (q)	1.35 (s, 3H) *ax* (α)	
G′1(→C3)	106.54 (d)	4.97 (d, *J* = 7.9 Hz, 1H)	
G′2	75.84 (d)	3.99 (m, 1H)	
G′3 ^a^	78.72 (d)	4.18 (m, 1H)	
G′4	73.23 (d)	4.19 (m, 1H)	
G′5	75.84 (d)	3.99 (m, 1H)	
G′6	69.10 (t)	4.50 (dd, *J* = 10.1, 4.1 Hz, 1H) A	4.22 (m, 1H) B
A″1(→G′6)	101.34 (d)	5.33 (d, *J* = 3.1 Hz, 1H)	
A″2	78.83 (d)	4.63 (m, 1H)	
A″3	72.00 (d)	4.65 (m, 1H)	
A″4	66.89 (d)	4.59 (m, 1H)	
A″5	62.72 (t)	4.39 (dd, *J* = 11.0, 7.9 Hz, 1H) A	3.92 (dd, *J* = 10.9, 3.8 Hz, 1H) B
G‴1(→A″2)	103.46 (d)	5.17 (d, *J* = 7.0 Hz, 1H)	
G‴2	78.93 (d)	4.18 (m, 1H)	
G‴3	79.65 (d)	4.16 (m, 1H)	
G‴4 ^b^	71.75 (d)	4.17 (m, 1H)	
G‴5	78.69 (d)	3.68 (m, 1H)	
G‴6	62.53 (t)	4.35 (dd, *J* = 12.1, 2.5 Hz, 1H) A	4.27 (dd, *J* = 13.2, 4.9 Hz, 1H) B
R″″1(→G‴2)	101.41 (d)	6.52 (d, *J* = 1.8 Hz, 1H)	
R″″2	83.14 (d)	4.78 (m, 1H)	
R″″3	73.01 (d)	4.66 (m, 1H)	
R″″4	75.11 (d)	4.23 (m, 1H)	
R″″5	69.99 (d)	4.85 (dd, *J* = 9.5, 6.3 Hz, 1H)	
R″″6	19.12 (q)	1.74 (d, *J* = 6.1 Hz, 3H)	
G‴″1(→R″″2)	107.82 (d)	5.25 (d, *J* = 7.9 Hz, 1H)	
G‴″2	76.26 (d)	4.06 (m, 1H)	
G‴″3 ^a^	78.74 (d)	4.18 (m, 1H)	
G‴″4 ^b^	71.84 (d)	4.17 (m, 1H)	
G‴″5	79.10 (d)	3.85 (m, 1H)	
G‴″6	63.07 (t)	4.43 (m, 1H) A	4.25 (m, 1H) B

^a,b^—assignments with the same letters may be exchanged.

**Table 2:** ^1^H-(600 MHz) and ^13^C-(125 MHz) NMR chemical shifts of compound **13** (in pyridine-*d5*).

Position	δ_C_ (ppm)	δ_H_ (ppm)	δ_H_ (ppm)
C1	36.26 (t)	1.68 (m, 1H) *eq*	1.18 (td, *J* = 13.6, 3.6 Hz, 1H) *ax*
C2	27.99 (t)	2.27 (m, 1H) *eq*	2.02 (m, 1H) *ax*
C3	89.43 (d)	3.57 (dd, *J* = 11.5, 4.6 Hz, 1H) *ax*	
C4	44.93 (s)	-	
C5	52.30 (d)	1.28 (dd, *J* = 12.7, 2.0 Hz, 1H) *ax*	
C6	19.25 (t)	1.83 (dd, *J* = 13.0, 6.5 Hz, 1H) *eq*	1.52 (m, 1H) *ax*
C7	27.41 (t)	2.02 (m, 2H)	
C8	135.79 (s)	-	
C9	135.12 (s)	-	
C10	37.30 (s)	-	
C11	21.56 (t)	2.12 (m, 1H) *eq*	1.95 (m, 1H) *ax*
C12	25.77 (t)	2.35 (m, 1H) *ax*	1.42 (m, 1H) *eq*
C13	49.34 (s)	-	
C14	51.32 (s)	-	
C15	32.67 (t)	1.67 (m, 1H) *ax*	1.40 (d, *J* = 2.6 Hz, 1H) *eq*
C16	42.28 (t)	1.99 (m, 1H) *ax*	1.74 (m, 1H) *eq*
C17	96.24 (s)	-	
C18	19.83 (q)	0.90 (s, 3H) *ax* (β)	
C19	20.03 (q)	0.94 (s, 3H) *ax* (β)	
C20	44.48 (d)	2.01 (m, 1H) *ax*	
C21	17.98 (q)	1.02 (d, *J* = 6.7 Hz, 3H) *eq*	
C22	41.01 (t)	1.68 (m, 2H)	
C23	74.13 (d)	4.61 (m, 1H) *ax*	
C24	53.31 (t)	2.85 (dd, *J* = 15.4, 7.8 Hz, 1H) A	2.61 (dd, *J* = 15.4, 5.7 Hz, 1H) B
C25	207.61 (s)	-	
C26	30.85 (q)	2.21 (s, 3H)	
*nor*-C27	-	-	
C28	23.66 (q)	1.56 (s, 3H) *eq*	
C29	63.67 (t)	4.44 (d, *J* = 11.2 Hz, 1H) A	3.62 (m, 1H) B
C30	26.86 (q)	1.35 (s, 3H) *ax* (α)	
G′1(→C3)	106.55 (d)	4.96 (d, *J* = 7.8 Hz, 1H)	
G′2	75.87 (d)	3.99 (m, 1H)	
G′3	78.77 (d)	4.21 (m, 1H)	
G′4	73.27 (d)	4.21 (m, 1H)	
G′5	75.87 (d)	4.02 (m, 1H)	
G′6	69.11 (t)	4.51 (dd, *J* = 10.2, 4.2 Hz, 1H) A	4.23 (dd, *J* = 10.4, 4.7 Hz, 1H) B
A″1(→G′6)	101.37 (d)	5.36 (d, *J* = 3.2 Hz, 1H)	
A″2	78.79 (d)	4.65 (m, 1H)	
A″3	71.97 (d)	4.68 (m, 1H)	
A″4	66.88 (d)	4.64 (m, 1H)	
A″5	62.66 (t)	4.41 (dd, *J* = 11.3, 8.1 Hz, 1H) A	3.95 (dd, *J* = 11.0, 4.0 Hz, 1H) B
G‴1(→A″2)	103.56 (d)	5.17 (d, *J* = 7.8 Hz, 1H)	
G‴2	78.10 (d)	4.27 (m, 1H)	
G‴3	79.92 (d)	4.18 (m, 1H)	
G‴4	71.84 (d)	4.21 (m, 1H)	
G‴5	78.74 (d)	3.70 (ddt, *J* = 6.9, 4.5, 2.3 Hz, 1H)	
G‴6	62.56 (t)	4.33 (m, 1H) A	4.26 (dd, *J* = 9.1, 7.5 Hz, 1H) B
R″″1(→G‴2)	102.43 (d)	6.39 (d, *J* = 1.8 Hz, 1H)	
R″″2	72.81 (d)	4.77 (dd, *J* = 3.5, 1.6 Hz, 1H)	
R″″3	73.12 (d)	4.66 (m, 1H)	
R″″4	74.72 (d)	4.31 (m, 1H)	
R″″5	70.17 (d)	4.92 (dd, *J* = 9.6, 6.2 Hz, 1H)	
R″″6	19.23 (q)	1.77 (d, *J* = 6.2 Hz, 3H)	

## Supplementary Materials

The following are available online, Figure S1: MS/MS fragmentation of compounds 12 and 13. Figure S2: 1D- and 2D-NMR spectra of 12. Figure S3: 1D- and 2D-NMR spectra of 13.

## Appendix A

**Table A1:** The list of Primulaceae species used for UHPLC-MS and -MS/MS screening in this study.

Taxon	Acronym	Taxon	Acronym
genus *Androsace* L.		genus *Cortusa* L.	
sct. ***Aretia***, ssct. ***Aretia***		*Cortusa matthioli*	CMAT_P_2013
***Androsace cylindrica***	**ACYL**_K_2015	*Cortusa matthioli* ssp. *matthioli*	CMAT_K_2015
*Androsace lehmannii*	ALEH_P_2014	*Cortusa matthioli* ssp. *caucasica*	CCAU_K_2015
***Androsace mathildae***	**AMTH**_TK_2015	*Cortusa matthioli* ssp. *sachalinensis*	CSAC_K_2015
sct. ***Aretia***, ssct. ***Dicranothrix***		*Cortusa matthioli* var. *sachalinensis*	CSAC_P_2013
***Androsace lactea***	**ALAC**_TK_2015	*Cortusa matthioli* ssp. *turkestanica*	CTUR_P_2013
***Androsace laggeri*** (= *A. carnea* var. *laggeri*)	**ACAL**_P_2014		
***Androsace obtusifolia***	**AOBT**_P_2014	genus *Dionysia* Fenzl.	
ct. *Chamaejasme*, ssct. *Hookerianae*		*Dionysia khatamii*	DKHA_F_2015
*Androsace limprichtii*	ALIM_P_2014	*Dionysia zschummelii*	DZSH_F_2015
sct. *Chamaejasme*, ssct. *Mucronifoliae*			
*Androsace mariae* var. *tibetica*	AMAT_P_2014	genus *Hottonia* L.	
*Androsace mucronifolia*	AMUC_TK_2015	*Hottonia inflata* ^hb^	HOIN_MO_2014
*Androsace sempervivoides*	ASPV_B_2015	*Hottonia palustris* ^hb^	HOPA_PG_2014
sct. *Chamaejasme*, ssct. *Strigillosae*			
*Androsace spinulifera*	ASPI_P_2015	genus *Soldanella* L.	
*Androsace strigillosa*	ASTR_P_2014	sct. *Crateriflorae*	
sct. *Chamaejasme*, ssct. *Sublanatae*		*Soldanella alpina*	SALP_K_2016
*Androsace adenocephala*	AADE_F_2015	*Soldanella carpatica*	SCAR_K_2014
*Androsace nortonii*	ANOR_P_2014	*Soldanella cyanaster*	SCYA_K_2014
sct. *Chamaejasme*, ssct. *Villosae*, series *Chamaejasmoidae*		*Soldanella dimoniei*	SDIM_K_2014
*Androsace brachystegia*	ABRA_P_2014	*Soldanella villosa*	SVIL_K_2014
*Androsace chamaejasme* ssp. *carinata*	ACHC_P_2014	sct. *Tubiflorae*	
*Androsace zambalensis*	AZAM_P_2014	*Soldanella minima*	SMIN_F_2015
sct. *Chamaejasme*, ssct. *Villosae*, series *Euvillosae*		*Soldanella minima*	SMIN_TK_2015
*Androsace dasyphylla*	ADAS_P_2014		
*Androsace robusta* ssp. *purpurea*	AROP_P_2014	genus ***Vitaliana*** Sesl.	
*Androsace sarmentosa*	ASAR_K_2015	***Vitaliana primuliflora***	**VPRI**_B_2015
sct. *Pseudoprimula*		***Vitaliana primuliflora*** **ssp. *assoana***	**VPAS**_TK_2015
*Androsace elatior*	AELA_F_2015	***Vitaliana primuliflora*** **ssp. *praetutiana***	**VPPR**_B_2015
sct. ***Douglasia***		***Vitaliana primuliflora*** **ssp. *praetutiana***	**VPPR**_B_2017
***Androsace******montana*** (= *Douglasia montana*)	**AMON**_F_2015		
sct. *Aizodium*			
*Androsace bulleyana*	ABUL_F_2015		

**^hb^**—herb was used instead of roots; **bold** names and abbreviations—samples abundant in newly described compounds **12** and **13**; sg.—subgenus, sct.—section, ssct.—subsection, ssp.—subspecies, var.—variety. The meaning of acronyms used to avoid long plant names in storage and during research is as follows: First letter—genus, three following letters—taxon (species; optionally together with variety), next separated letter or two—donator abbreviation and year of collection in the end. Donators abbreviations: B—Bergenia, Nursery, Paweł Weinar, Kokotów, Poland; F—Floralpin, Nursery, Frank Schmidt, Waldenbuch, Germany; K—Kevock Garden, Nursery, Stella and David Rankin, Lasswade, UK; MO—Mayla Ogrody, Nursery, Dawid Stefaniuk, Siedlakowice, Poland; P—Josef and Bohumila Plocar, Nursery, Švihov, Czech Republic; PG—Planta Garden, Krzysztof Sternal, Dobra, Poland; TK—Private Collection, Tomasz Kubala, Poland.

**Table A2:** UHPLC-MS and detailed -MS/MS results for *Vitaliana primulifora* samples.

						Sample Abbreviations	VPPR_B_15	VPAS_TK_15	VPRI_B_15
**No.**	**RT ± RTSD** **[min]**	**Proposed Neutral Formula**	**calcd. *m*/*z*** **[for Neut. Form.–H]^−^**	**|Error|** **[ppm]**	**BPC Fragments;** **Collision Energy 30 eV** **(Relative Intensity %)**	**MS2 Fragments** **/Collision Energy/** **(Relative Intensity %)**	**Rel. Area %**	**Rel. Area %**	**Rel. Area %**
		^1^ glycoside ^2^ aglycone			**[suggested interpretation]**	**[suggested interpretation]**			
1.	6.82 ± 0.01	C_58_H_98_O_27_ C_29_H_50_O_4_	1225.6223 461.3636	2.8 2.7	1339.6070 (2.1) [M+FNa+FA–H]^−^ 1293.6067 (8.3) [M+FNa–H]^−^ **1225.6188 (100) [M–H]^−^**	/96.0 eV/ **1079.5619 (49.7) [M–dxHex–H]^−^** **917.5039 (100) [M–dxHex–Hex–H]^−^** 785.4706 (38.5) [M–dxHex–Hex–Pen–H]^−^ **623.4139 (98.1) [M–dxHex–Hex–Pen–Hex–H]^−^** 461.3649 (4.7) [M–dxHex–Hex–Pen–Hex–Hex–H]^−^ 367.1239 (29.5) [M–dxHex–Hex–Pen–Hex–256–H]^−^	14.31	9.60	0.00
2.	7.38 ± 0.01	C_53_H_86_O_24_ C_37_H_44_O	1105.5436 503.3319	2.4 13.7	1173.5263 (4.7) [M+FNa–H]^−^ **1105.5410 (100) [M–H]^−^**	/86.4 eV/ **1105.54 (100) [M–H]^−^** 959.4814 (21.9) [M–dxHex–H]^−^ 941.4744 (4.2) [M–dxHex–18–H]^−^ 797.4326 (8.4) [M–dxHex–Hex–H]^−^ 779.4224 (4.6) [M–dxHex–Hex–18–H]^−^ 665.3906 (1.6) [M–dxHex–Hex–Pen–H]^−^ 647.383 (1) [M–dxHex–Hex–Pen–18–H]^−^ 503.3388 (2.1) [M–dxHex–Hex–Pen–Hex–H]^−^	10.52	8.06	0.00
3.	7.82 ± 0.01	C_58_H_94_O_28_ C_29_H_46_O_5_	1237.5859 473.3272	1.1 5.2	1351.5710 (6.3) [M+FA+FNa–H]^−^ 1305.5669 (7.2) [M+FNa–H]^−^ 1283.5881 (30.1) [M+FA–H]^−^ **1237.5845 (100) [M–H]^−^**	/97.0 eV/ 1237.5700 (5.0) [M–H]^−^ 1075.5204 (15.4) [M–Hex–H]^−^ **929.4692 (57.0) [M–Hex–dxHex–H]^−^** **767.4243 (80.9) [M–Hex–dxHex–Hex–H]^−^** 655.3349 (11.8) 635.3776 (19.0) [M–Hex–dxHex–Hex–Pen–H]^−^ 529.1791 (20.9) 473.3248 (14.9) [M–Hex–dxHex–Hex–Pen–Hex–H]^−^ 395.1227 (23.2) [M–Hex–dxHex–Hex–Pen–240–H]^−^ **367.1257 (100) [M–Hex–dxHex–Hex–Pen–268–H]^−^** 293.0879 (20.7)	7.51	8.01	9.00
4.	8.07 ± 0.00	C_52_H_84_O_23_ C_29_H_46_O_5_	1075.533 1473.3272	0.9 10.2	1189.5237 (7.5) [M+FA+FNa–H]^−^ 1143.5171 (5.8) [M+FNa–H]^−^ 1121.5363 (27.7) [M+FA–H]^−^ **1075.5321 (100) [M–H]^−^**	/84.0 eV/ 1075.5673 (3.1) [M–H]^−^ **929.4713 (55.5) [M–dxHex–H]^−^** **767.4235 (100) [M–dxHex–Hex–H]^−^** 655.3402 (15.7) 635.3733 (19.6) [M–dxHex–Hex–Pen–H]^−^ 541.1746 (10.0) 473.3321 (24.7) [M–dxHex–Hex–Pen–Hex–H]^−^ 395.1206 (21.8) 367.1247 (43.1) [M–dxHex–Hex–Pen–Hex–106–H]^−^ 361.237 (18.2)	6.32	7.55	6.11
5.	8.38 ± 0.01	C_53_H_84_O_24_ C_29_H_44_O_4_	1103.5280 455.3167	1.0 3.1	1171.5138 (7.5) [M+FA+FNa–H]^−^ **1103.5269 (100) [M+FA–H]^−^**	/86.3 eV/ **1105.5379 (66.1) [M+FA+2–H]^−^** **1103.5269 (30.8) [M+FA–H]^−^** **1057.5203 (100) [M–H]^−^** 959.4818 (14.1) 957.4602 (11.6) [911+FA]^−^ 913.4669 (22.6) [911+2]^−^ 911.4629 (46.5) [M–dxHex–H]^−^ 893.455 (8.1) 751.4255 (32.4) [749+2]^−^ 749.4088 (23.1) [M–dxHex–Hex–H]^−^ 731.4039 (7.5) 705.4239 (9.4) 639.3456 (13.7) 617.3689 (10) [M–dxHex–Hex–Pen–H]^−^ 541.1791 (5.3) 457.3345 (13.3) [455+2]^−^ 455.3181 (17.7) [M–dxHex–Hex–Pen–Hex–H]^−^ 395.1199 (12.1) 367.1246 (22.6) [M–dxHex–Hex–Pen–Hex–88–H]^−^ 353.1113 (5.3) 345.244 (9.6) 293.0849 (10.4)	6.58	5.41	0.00
6.	8.57 ± 0.00	C_57_H_90_O_28_ n.i.	1221.5546	3.2	1335.5407 (5.1) [M+FA+FNa–H]^−^ 1289.5337 (7.0) [M+FNa–H]^−^ 1267.5545 (12.8) [M+FA–H]^−^ **1221.5507 (100) [M–H]^−^**	not fragmented	2.82	3.22	3.35
7.	8.65 ± 0.00	C_58_H_92_O_28_ C_29_H_44_O_5_	1235.5702 471.3116	0.6 1.3	1349.5618 (6.3) [M+FA+FNa–H]^−^ 1303.5527 (5.3) [M+FNa–H]^−^ 1281.5739 (37.0) [M+FA–H]^−^ **1235.5695 (100) [M–H]^−^**	/96.8 eV/ 1235.5841 (1.7) [M–H]^−^ 1217.5749 (4.6) [M–18–H]^−^ 1177.5321 (3.8) [M–18–40–H]^−^ 1073.5132 (3.9) [M–Hex–H]^−^ 1055.5035 (8.7) [M–Hex–18–H]^−^ 1015.4795 (10.2) [M–Hex–18–40–H]^−^ 927.4543 (31.3) [M–Hex–dxHex–H]^−^ **909.4439 (49.5) [M–Hex–dxHex–18–H]^−^** 869.4148 (36.8) [M–Hex–dxHex–18–40–H]^−^ **765.4053 (67.7) [M–Hex–dxHex–Hex–H]^−^** **747.3951 (78.8) [M–Hex–dxHex–Hex–18–H]^−^** **707.3648 (46.1) [M–Hex–dxHex–Hex–18–40–H]^−^** 633.3663 (26.1) [M–Hex–dxHex–Hex–Pen–H]^−^ 615.3533 (19.5) [M–Hex–dxHex–Hex–Pen–18–H]^−^ 575.3221 (12.7) [M–Hex–dxHex–Hex–Pen–18–40–H]^−^ 529.1771 (17.2) 471.3122 (9.9) [M–Hex–dxHex–Hex–Pen–Hex–H]^−^ 469.1568 (9.2) [M–Hex–dxHex–Hex–Pen–Hex–2–H]^−^ 453.3017 (37.1) 413.2702 (29.1) 395.1191 (18.5) **367.1253 (100)** 353.1075 (6.8) 307.1017 (5.4) 293.0873 (14.9)	22.43	41.90	30.37
8.	8.99 ± 0.01	C_52_H_82_O_23_ C_29_H_44_O_5_	1073.5174 471.3116	3.1 0.4	1187.5045 (6.4) [M+FA+FNa–H]^−^ 1141.5003 (4.3) [M+FNa–H]^−^ 1119.5172 (21.1) [M+FA–H]^−^ **1073.5141 (100) [M–H]^−^**	/83.9 eV/ 1073.5027 (2.0) [M–H]^−^ 1015.4776 (2.0) [M–18–40–H]^−^ 927.4560 (29.9) [M–dxHex–H]^−^ 909.4401 (17.3) [M–dxHex–18–H]^−^ 869.4096 (19.0) [M–dxHex–18–40–H]^−^ **765.4025 (100) [M–dxHex–Hex–H]^−^** 747.3931 (44.1) [M–dxHex–Hex–18–H]^−^ 707.3619 (34.7) [M–dxHex–Hex–18–40–H]^−^ **633.3613 (47.8) [M–dxHex–Hex–Pen–H]^−^** 615.3514 (17.2) [M–dxHex–Hex–Pen–18–H]^−^ 575.3216 (8.7) [M–dxHex–Hex–Pen–18–40–H]^−^ 471.3114 (15.2) [M–dxHex–Hex–Pen–Hex–H]^−^ 453.3007 (27.3) [M–dxHex–Hex–Pen–Hex–18–H]^−^ 413.2691 (25.6) [M–dxHex–Hex–Pen–Hex–18–40–H]^−^ 395.1204 (12.5) 367.1240 (26.8) 353.1086 (7.9) 293.0858 (13.8)	22.46	42.64	28.55
9.	11.56 ± 0.00	C_58_H_94_O_28_ C_29_H_46_O_5_	1237.5859 473.3272	2.5 1.0	1351.5736 (4.8) [M+FA+FNa–H]^−^ 1305.5669 (7.2) [M+FNa–H]^−^ 1283.5868 (18.2) [M+FA–H]^−^ **1237.5828 (100) [M–H]^−^**	/97.0 eV/ 1237.5901 (6) [M–H]^−^ 1075.5277 (10.4) [M–Hex–H]^−^ **929.4714 (50) [M–Hex–dxHex–H]^−^** **767.4205 (58.6) [M–Hex–dxHex–Hex–H]^−^** 703.2274 (7.7) 661.2146 (26.9) 635.3797 (17.4) [M–Hex–dxHex–Hex–Pen–H]^−^ 541.1773 (9.4) 529.1758 (31.1) 499.166 (40.3) 473.3268 (18.6) [M–Hex–dxHex–Hex–Pen–Hex–H]^−^ 395.1193 (18.3) **367.1245 (100) [M–Hex–dxHex–Hex–Pen–Hex–106–H]^−^** 353.1087 (55.4) 293.0868 (9.4)	17.30	0.00	3.56
10.	12.08 ± 0.00	C_52_H_84_O_23_ C_29_H_46_O_5_	1075.5331 473.3272	3.7 1.0	1189.5223 (8.8) [M+FA+FNa–H]^−^ 1143.5168 (6.3) [M+FNa–H]^−^ **1121.5355 (61.4) [M+FA–H]^−^** **1075.5291 (100) [M–H]^−^**	/84.0 eV/ 1075.5302 (5.3) [M–H]^−^ **929.4737 (58.4) [M–dxHex–H]^−^** **767.4192 (86.9) [M–dxHex–Hex–H]^−^** 635.3783 (32.2) [M–dxHex–Hex–Pen–H]^−^ 541.172 (13) 499.1677 (50.6) [M–dxHex–Hex–Pen–Pen+4–H]^−^ 473.3265 (42.2) [M–dxHex–Hex–Pen–Hex–H]^−^ 395.1217 (36.2) **367.1258 (68.6) [M–dxHex–Hex–Pen–Hex–106–H]^−^** **353.1086 (100) [M–dxHex–Hex–Pen–Pen+4–dxHex–H]^−^** 335.0984 (16) 293.087 (13.1)	6.27	0.00	0.00
11.	13.81 ± 0.01	C_57_H_92_O_27_ n.i.	1207.5753	4.8	1321.5589 (4.1) [M+FA+FNa–H]^−^ 1275.5544 (7.6) [M+FNa–H]^−^ 1253.5718 (11.6) [M+FA–H]^−^ **1207.5695 (100) [M–H]^−^**	/94.6 eV/ **913.4668 (100) [M–(Hex+Pen)–H]^−^**	10.26	4.89	8.53
12.	13.98 ± 0.00	C_58_H_94_O_27_ C_29_H_26_O_4_	1221.5910 457.3323	1.7 2.9	1335.5780 (4.3) [M+FA+FNa–H]^−^ 1289.5731 (5.0) [M+FNa–H]^−^ 1267.5928 (31.9) [M+FA–H]^−^ **1221.5889 (100) [M–H]^−^**	/95.7 eV/ 1221.5829 (7.7) [M–H]^−^ 1059.5321 (15.7) [M–Hex–H]^−^ **913.4762 (79.4) [M–Hex–dxHex–H]^−^** 895.4661 (6.6) **751.4257 (100) [M–Hex–dxHex–Hex–H]^−^** 619.3837 (20) [M–Hex–dxHex–Hex–Pen–H]^−^ 541.1739 (4.7) 529.1764 (15.5) 469.1561 (8.1) 457.3310 (7.9) [M–Hex–dxHex–Hex–Pen–Hex–H]^−^ 439.1443 (3.9) 395.1182 (16.7) **367.1242 (82.1) [M–Hex–dxHex–Hex–Pen–Hex–90–H]^−^** 353.1066 (5.4) 345.2439 (3.8) 293.0871 (11.4)	100.00	100.00	100.00
13.	14.69 ± 0.02	C_52_H_84_O_22_ C_29_H_26_O_4_	1059.5381 457.3323	2.1 1.7	1173.5257 (5.8) [M+FA+FNa–H]^−^ 1127.5206 (4.2) [M+FNa–H]^−^ 1105.5401 (31.4) [M+FA–H]^−^ **1059.5359 (100) [M–H]^−^**	/82.8 eV/ 1059.5390 (4.5) [M–H]^−^ **913.4783 (50) [M–dxHex–H]^−^** **751.427 (100) [M–dxHex–Hex–H]^−^** 619.3838 (23.3) [M–dxHex–Hex–Pen–H]^−^ 457.3331 (12.3) [M–dxHex–Hex–Pen–Hex–H]^−^ 395.1209 (13.8) 367.1245 (24.6) [M–dxHex–Hex–Pen–Hex–90–H]^−^ 293.0866 (13.2)	50.19	30.06	30.30

M—glycoside neutral mass (based on comparison spectra in 10 and 30 eV), FA—formic acid neutral mass, FNa—sodium formate neutral mass, Hex—hexose (loss), dxHex—deoxyhexose (loss), Pen—pentose (loss). Bold MS fragment—exceeding 50% of relative intensity. Underlined MS^2^ fragment—expected deprotonated aglycone ion. RT_SD_ based on triplicate measurement. n.i.—not identified due to lack of reliable fragmentation.

## References

[1] Challinor V.L., de Voss J.J. (2013). Open-chain steroidal glycosides, a diverse class of plant saponins. Nat. Prod. Rep..

[2] Vermaak I., Hamman J., Viljoen A. (2011). *Hoodia gordonii*: An up-to-date review of a commercially important anti-obesity plant. Planta Med..

[3] Gauthaman K., Ganesan A.P. (2008). The hormonal effects of *Tribulus terrestris* and its role in the management of male erectile dysfunction—an evaluation using primates, rabbit and rat. Phytomedicine.

[4] Adinolfi M., Corsaro M.M., Lanzetta R., Mancino A., Mangoni L., Parrilli M. (1993). Triterpenoid oligoglycosides from *Chionodoxa luciliae*. Phytochemistry.

[5] Siddiqi M.H., Siddiqi M.Z., Ahn S., Kang S., Kim Y.-J., Sathishkumar N., Yang D.-U., Yang D.-C. (2013). Ginseng saponins and the treatment of osteoporosis: Mini literature review. J. Ginseng Res..

[6] Keller A.-C., Keller J., Maillard M.P., Hostettmann K. (1997). A lanostane-type steroid from the fungus *Ganoderma carnosum*. Phytochemistry.

[7] Hostettmann K., Marston A. (1995). Saponins.

[8] Nes W.D. (2011). Biosynthesis of cholesterol and other sterols. Chem. Rev..

[9] Buckingham J., Cooper C.M., Purchase R. (2016). Natural products desk reference.

[10] Fan M.-Y., Wang Y.-M., Wamng Z.-M., Gao H.-M. (2014). Advances on chemical constituents and pharmacological activity of genus *Scilla*. China J. Chinese Mater. Medica.

[11] Wang G.-W., Lu C., Yuan X., Ye J., Jin H.-Z., Shan L., Xu X.-K., Shen Y.-H., Zhang W.-D. (2015). Lanostane-type triterpenoids from *Abies faxoniana* and their DNA topoisomerase inhibitory activities. Phytochemistry.

[12] Kalinin V.I., Avilov S.A., Silchenko A.S., Stonik V.A. (2015). Triterpene glycosides of sea cucumbers (Holothuroidea, Echinodermata) as taxonomic markers. Nat. Prod. Commun..

[13] Silchenko A.S., Kalinovsky A.I., Avilov S.A., Andryjaschenko P.V., Dmitrenok P.S., Yurchenko E.A., Ermakova S.P., Malyarenko O.S., Dolmatov I.Y., Kalinin V.I. (2018). Cladolosides C4, D1, D2, M, M1, M2, N and Q, new triterpene glycosides with diverse carbohydrate chains from sea cucumber *Cladolabes schmeltzii*. An uncommon 20,21,22,23,24,25,26,27-okta-nor-lanostane aglycone. The synergism of inhibitory action of non-toxic dose of the glycosides and radioactive irradiation on colony formation of HT-29 cancer cells. Carbohydr. Res..

[14] Ríos J.-L., Andújar I., Recio M.-C., Giner R.-M. (2012). Lanostanoids from fungi: A group of potential anticancer compounds. J. Nat. Prod..

[15] Xia Q., Zhang H., Sun X., Zhao H., Wu L., Zhu D., Yang G., Shao Y., Zhang X., Mao X. (2014). A comprehensive review of the structure elucidation and biological activity of triterpenoids from *Ganoderma* spp.. Molecules.

[16] Colombo P.S., Flamini G., Rodondi G., Giuliani C., Santagostini L., Fico G. (2017). Phytochemistry of European *Primula* species. Phytochemistry.

[17] Yamada Y., Hagiwara K., Iguchi K., Suzuki S., Hsu H. (1978). Isolation and structures of arvenins from *Anagallis arvensis* L. (*Primulaceae*). New cucurbitacin glucosides. Chem. Pharm. Bull..

[18] Budzianowski J., Morozowska M., Wesołowska M. (2005). Lipophilic flavones of *Primula veris* L. from field cultivation and in vitro cultures. Phytochemistry.

[19] Valant-Vetschera K.M., Bhutia T.D., Wollenweber E. (2009). Exudate flavonoids of *Primula* spp: Structural and biogenetic chemodiversity. Nat. Prod. Commun..

[20] Frankiewicz A.N. (2016). The analysis of saponins in some species of the *Primulaceae* Batsch. Master’s Thesis.

[21] Włodarczyk M., Matysik G., Cisowski W., Gleńsk M. (2006). Rapid densitometric quantitative screening of the myricitrin content of crude methanolic extracts of leaves from a variety of *Acer* species. J. Planar Chromatogr..

[22] Markham K.R. (1982). Techniques of Flavonoids Identification.

[23] Kuroda M., Mimaki Y., Ori K., Sakagami H., Sashida Y. (2004). 27-norlanostane glycosides from the bulbs of *Muscari paradoxum*. J. Nat. Prod..

[24] Adinolfi M., Barone G., Corsaro M.M., Mangoni L., Lanzetta R., Parrilli M. (1988). Glycosides from *Muscari armeniacum* and *Muscari botryoides*. Isolation and structure of Muscarosides G-N. Can. J. Chem..

[25] Sholichin M., Miyahara K., Kawasaki T. (1985). Oligoglycosides of spirocyclic nortriterpenoids related to eucosterol. Chem. Pharm. Bull..

[26] Wang L.-K., Zheng C.-J., Li X.-B., Chen G.-Y., Han C.-R., Chen W.-H., Song X.-P. (2014). Two new lanostane triterpenoids from the branches and leaves of *Polyalthia oblique*. Molecules.

[27] Smith G., Lowe D. (1997). The genus Androsace: A monograph for gardeners and botanists.

[28] Li G., Kusari S., Kusari P., Kayser O., Spiteller M. (2015). Endophytic *Diaporthe* sp. LG23 produces a potent antibacterial tetracyclic triterpenoid. J. Nat. Prod..

