# Supplementary material for: Lanostane-Type Saponins from Vitaliana primuliflora
Source: Molecules. 2019 Apr 23;24(8):1606. doi: 10.3390/molecules24081606 (PMC6515439; doi:10.3390/molecules24081606)
Supplement: Supplementary file 1 [file molecules-24-01606-s001.pdf]

Supplementary Materials

Lanostane-Type Saponins from *Vitaliana primuliflora*

Maciej Włodarczyk [1,*], Antoni Szumny [2] and Michał Gleńsk [1]

1 Department of Pharmacognosy and Herbal Medicines, Faculty of Pharmacy with Division of Laboratory Diagnostics, Wroclaw Medical University; Borowska 211a, 50-556 Wroclaw, Poland;
2 Department of Chemistry, Faculty of Food Science, Wrocław University of Environmental and Life Sciences; Norwida 25, 50-375 Wroclaw, Poland;
* Correspondence: maciej.wlodarczyk@umed.wroc.pl; Tel.: +48-71-78-40-223

Figure S1: MS/MS fragmentation of compounds **12** and **13**. H for loss of hexose, dH for loss of deoxyhexose, P for loss of pentose.

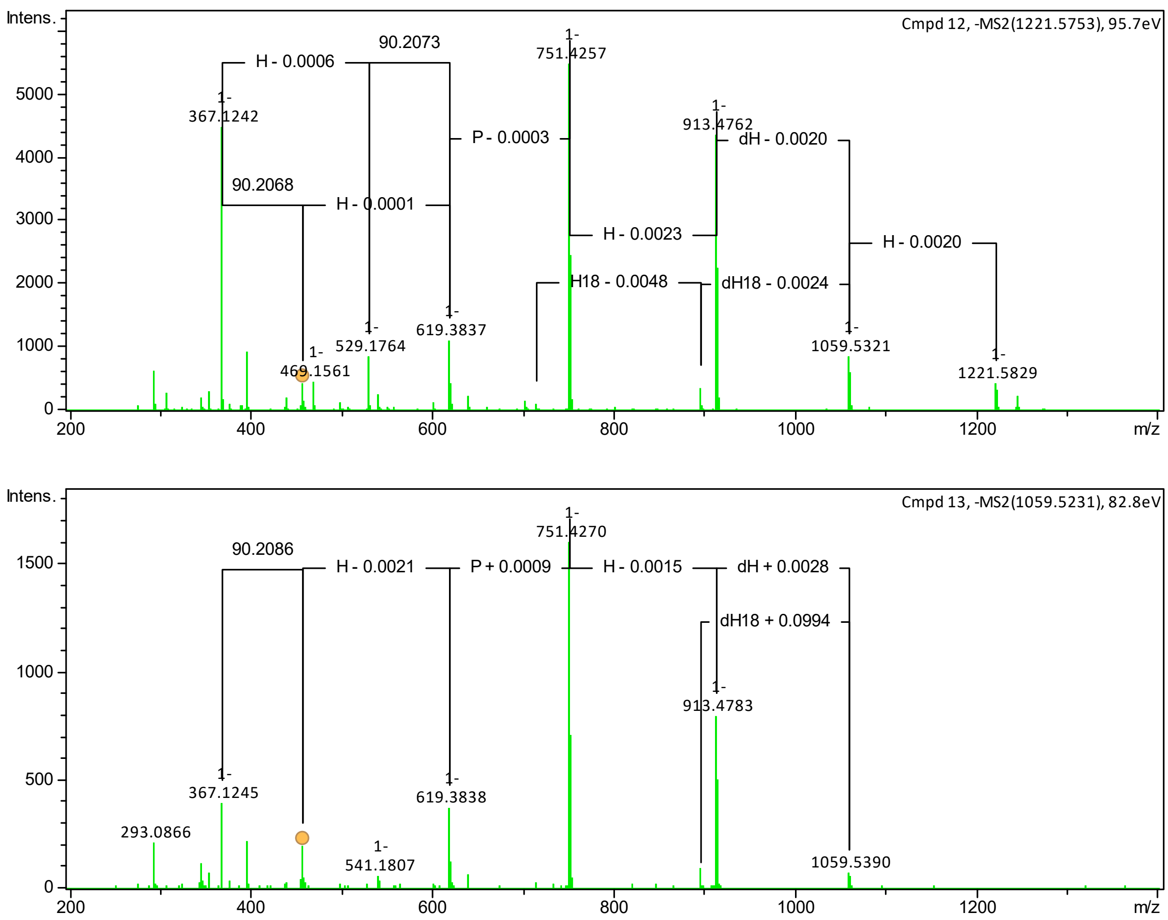

Figure S2: 1D and 2D NMR spectrum of **12**: ^{1}H, ^{13}C, HSQC, HMBC, TOCSY, NOESY.

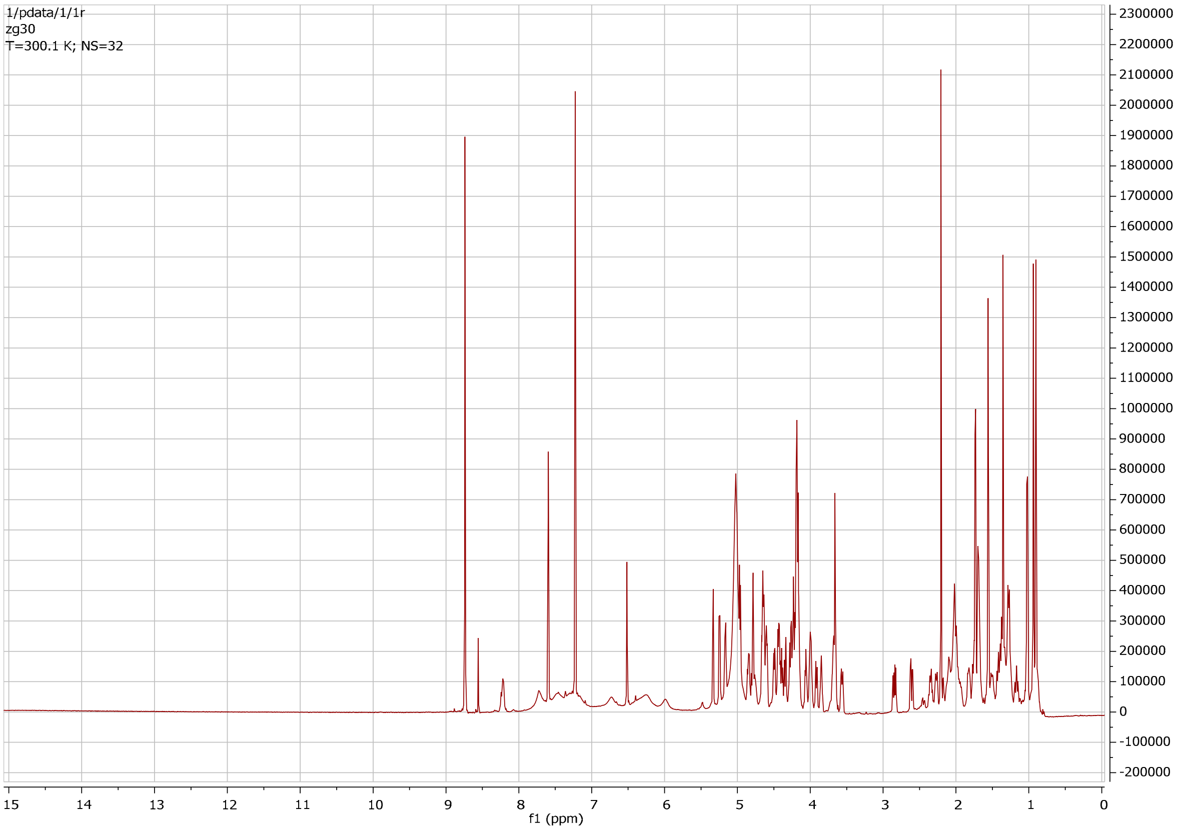

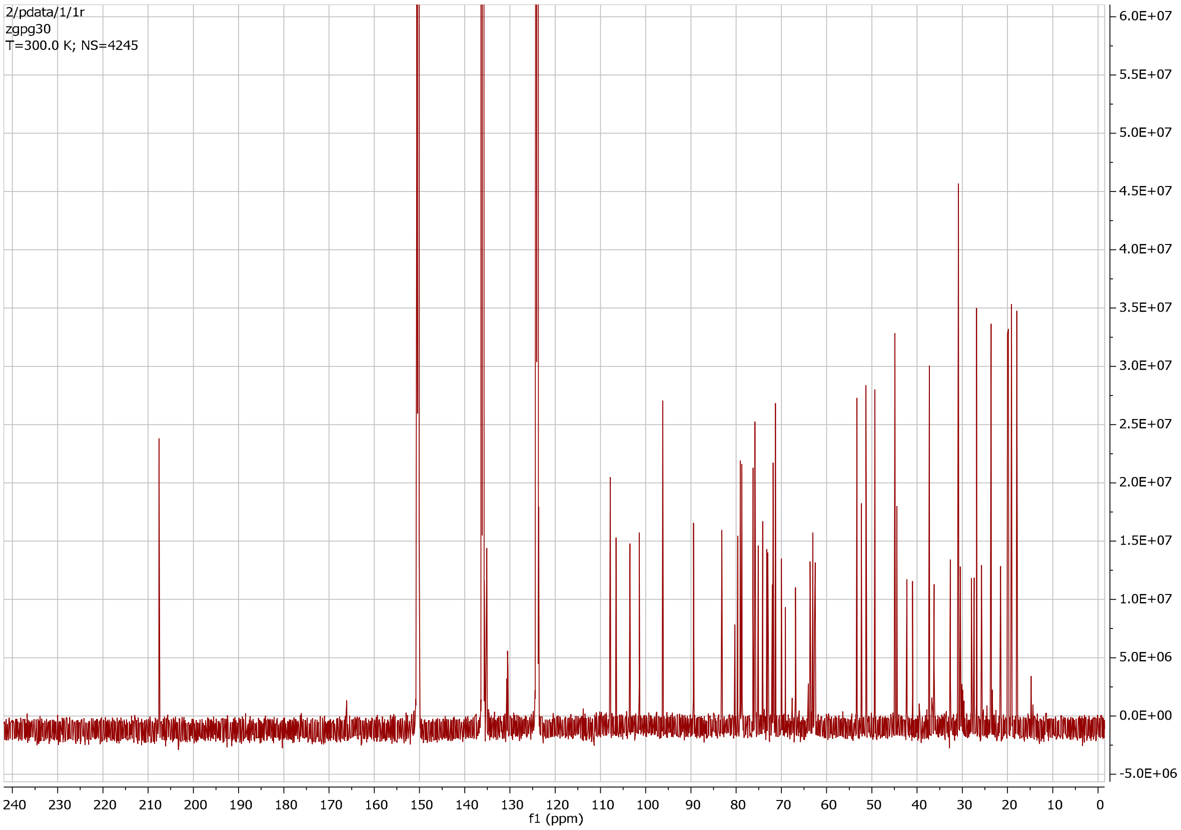

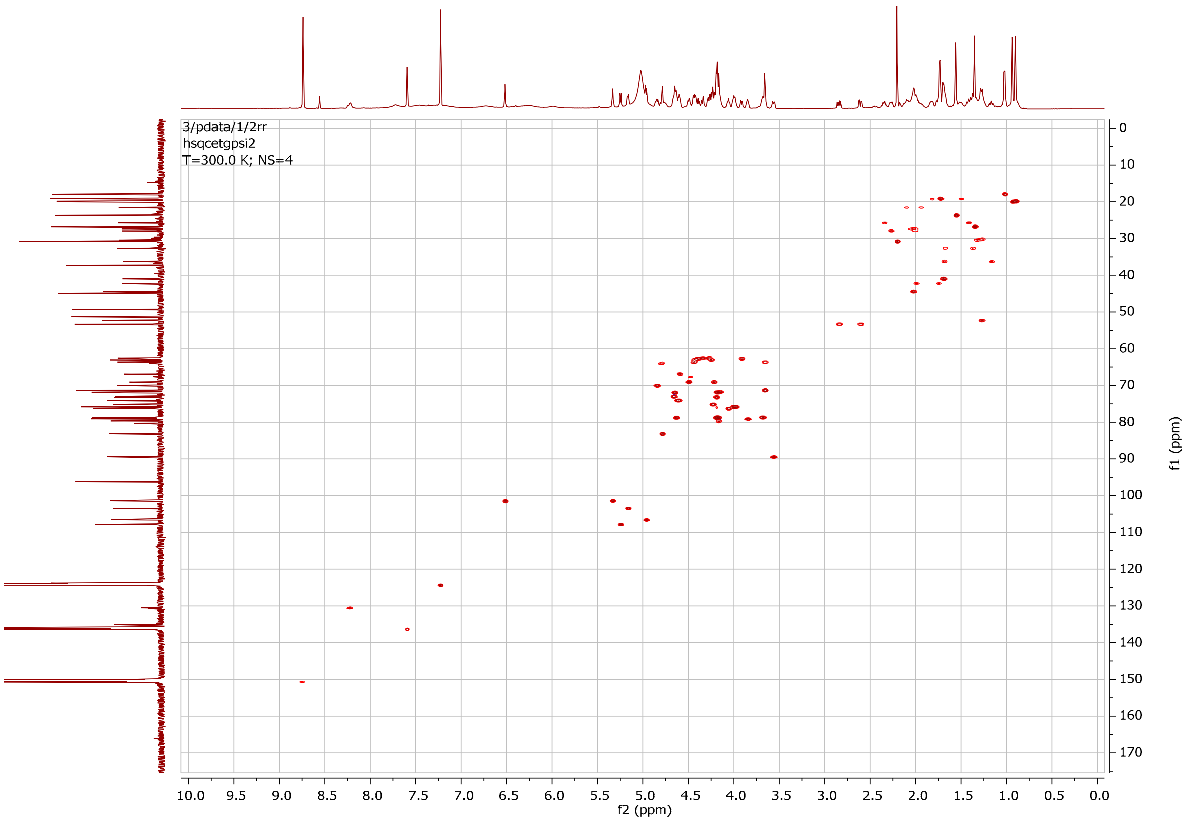
3/pdata/1/2rr
hsqcetgpsi2
T=300.0 K; NS=4
f2 (ppm)
f1 (ppm)

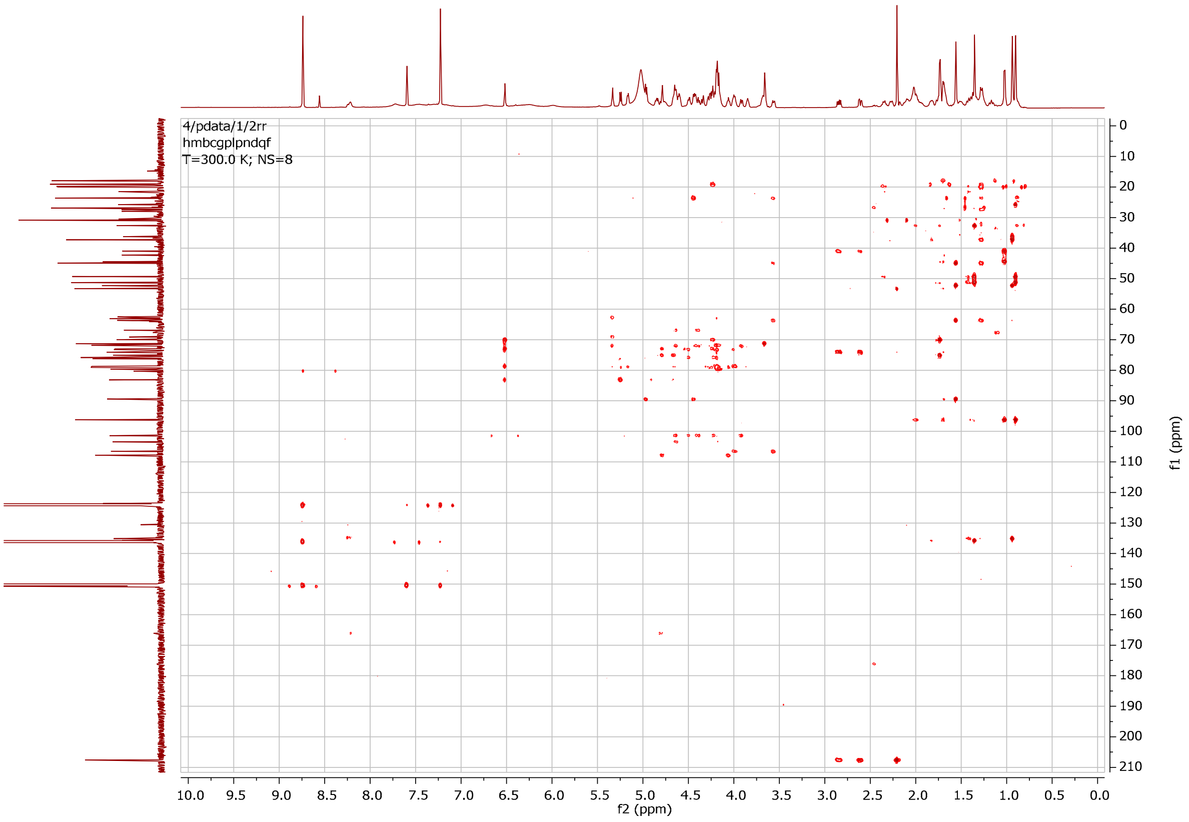
4/pdata/1/2rr
hmbcgplpndqf
T=300.0 K; NS=8
f2 (ppm)
f1 (ppm)

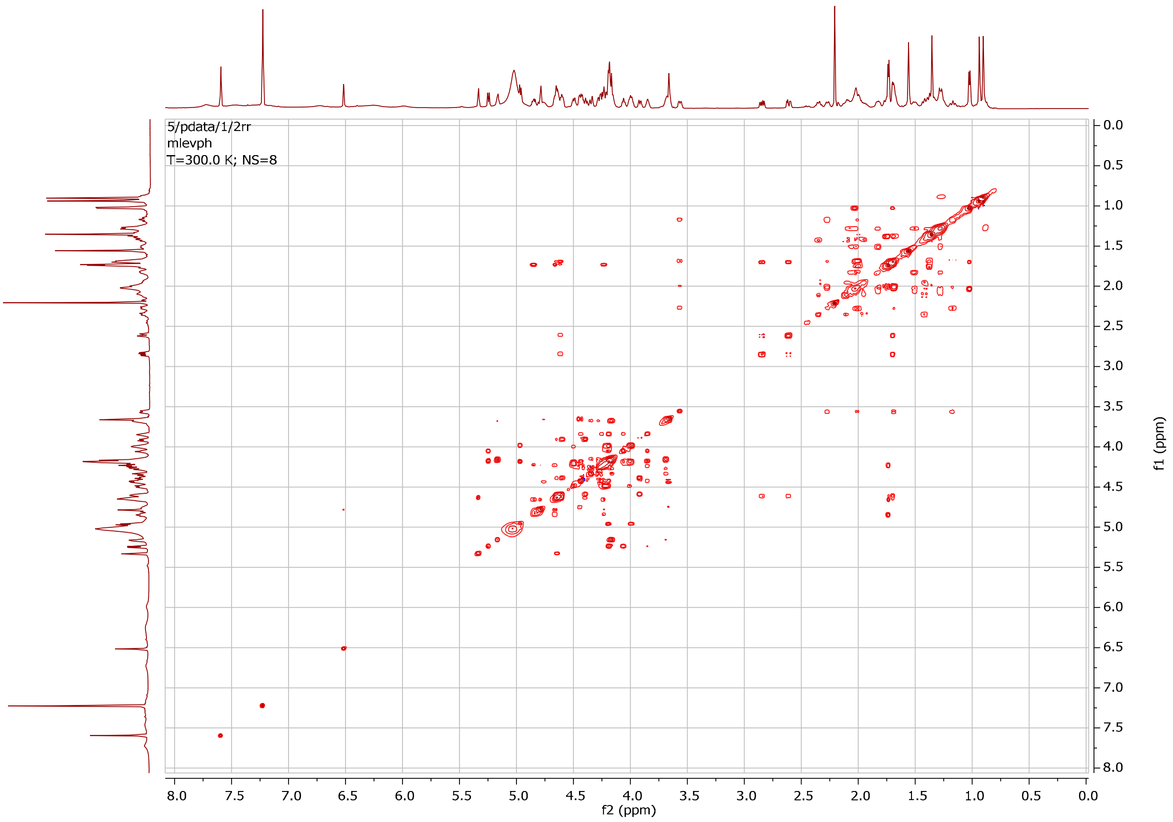
5/pdata/1/2rr
mlevph
T=300.0 K; NS=8
f2 (ppm)
f1 (ppm)

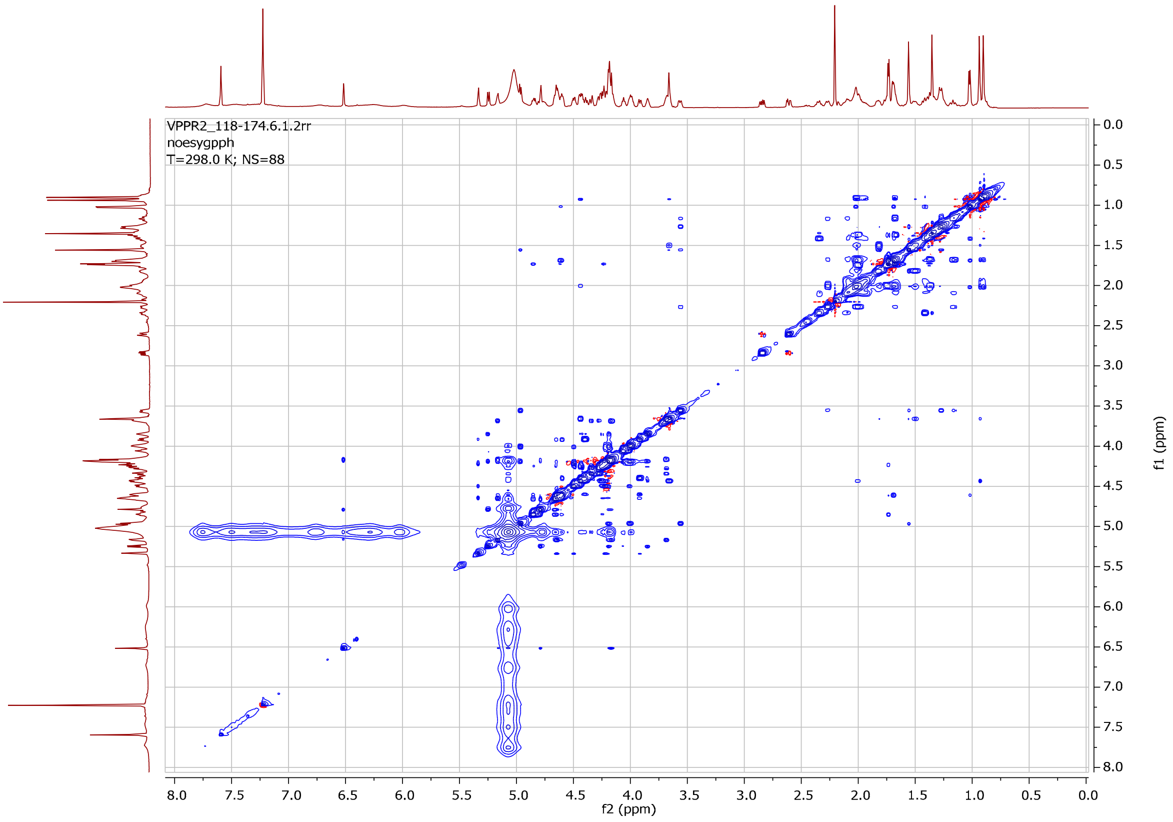
VPPR2_118-174.6.1.2rr
noesygpph
T=298.0 K; NS=88
f2 (ppm)
f1 (ppm)

Figure S3: 1D and 2D NMR spectrum of **13**: ^{1}H, ^{13}C, HSQC, HMBC, TOCSY, NOESY.

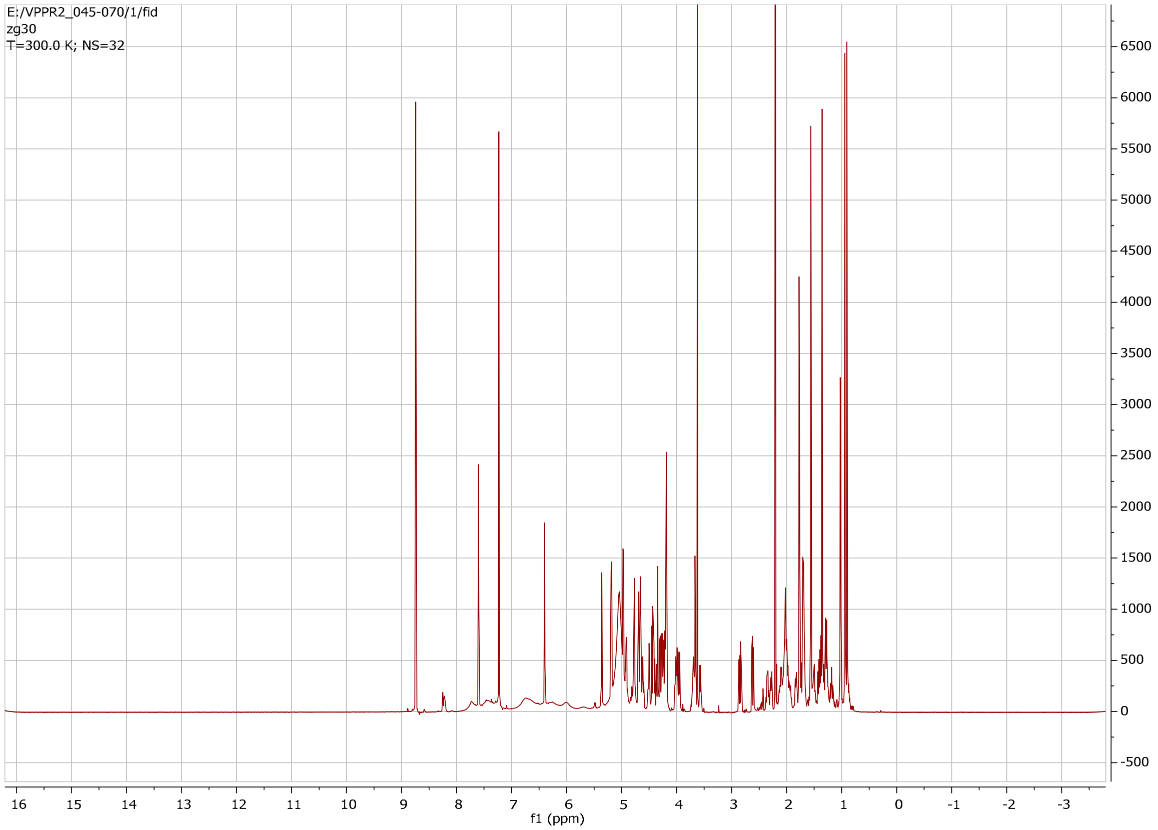

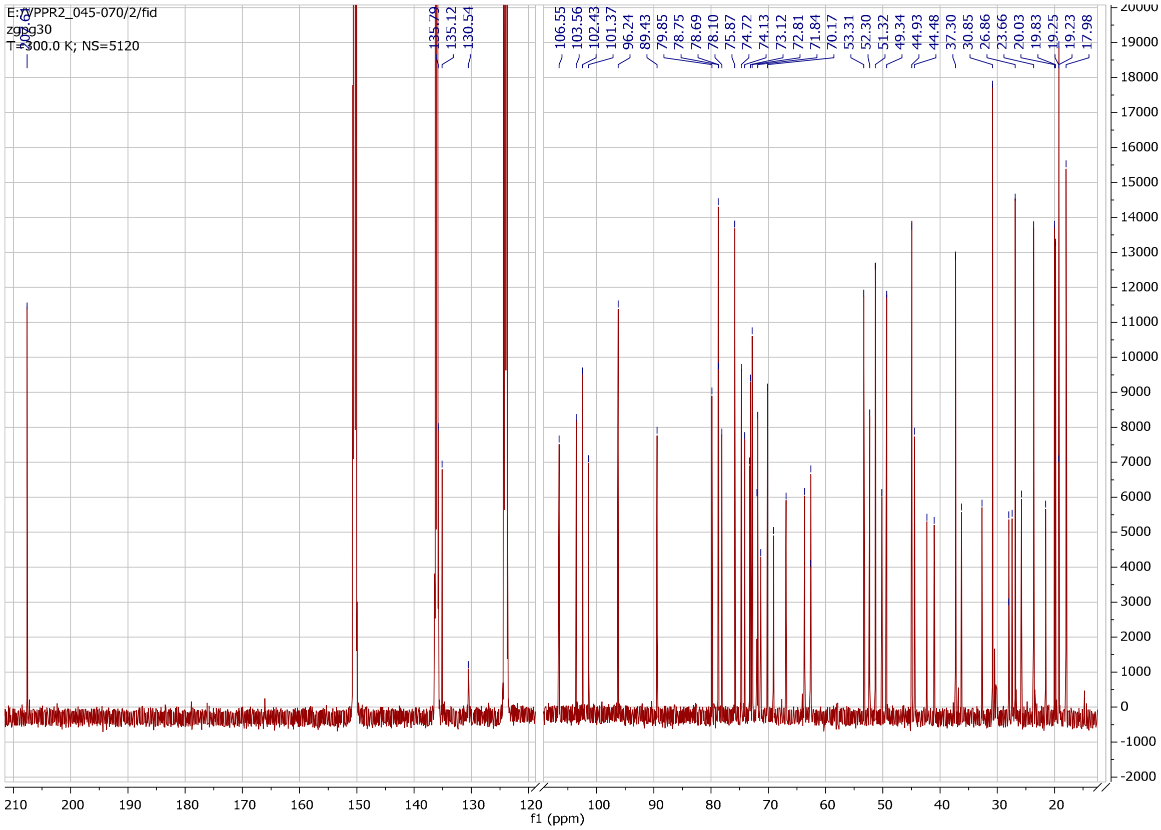

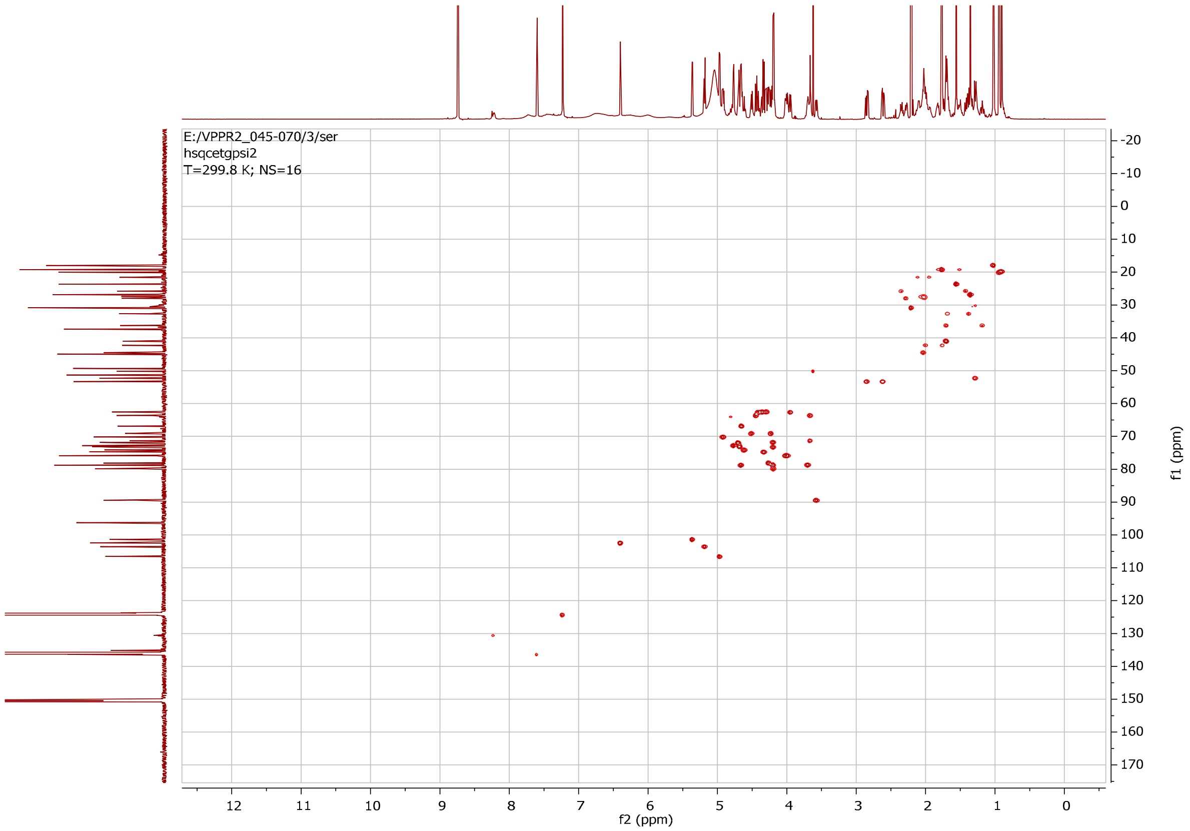
E:/VPPR2_045-070/3/ser
hsqcetgpsi2
T=299.8 K; NS=16
f2 (ppm)
f1 (ppm)

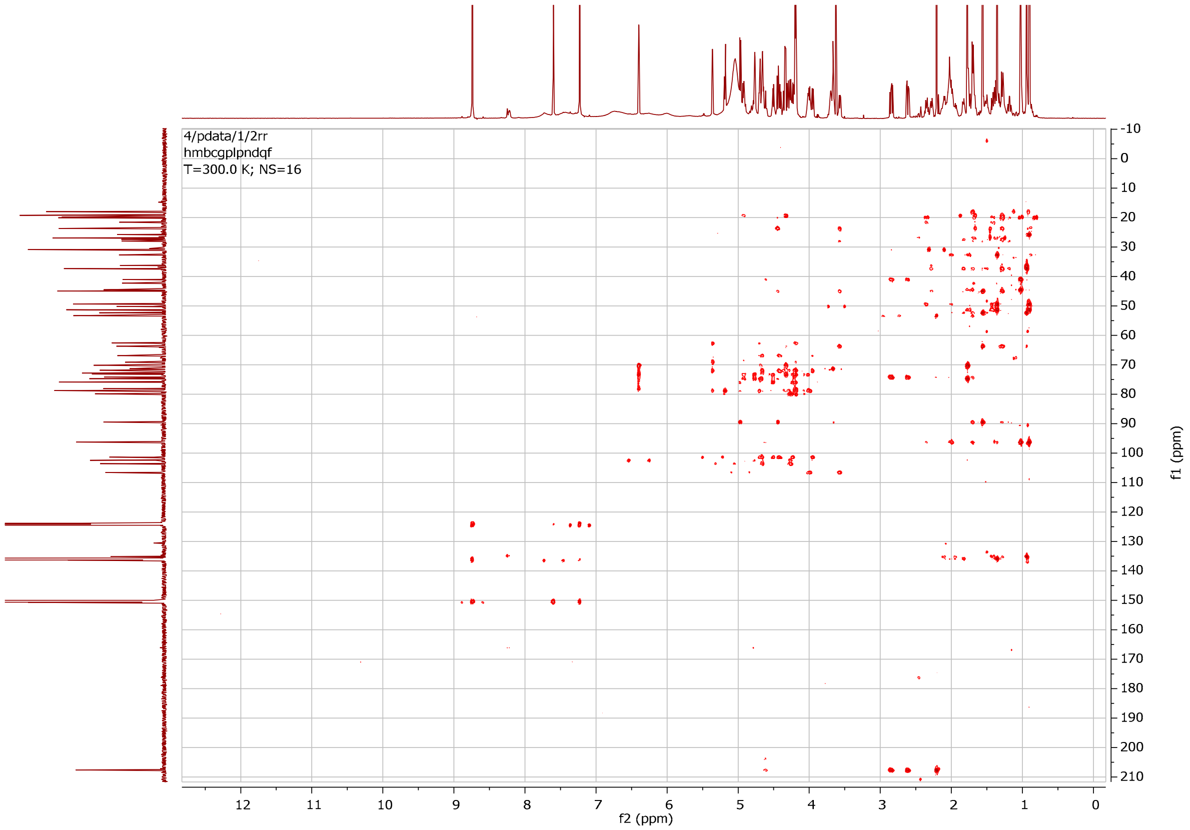
4/pdata/1/2rr
hmbcgplpndqf
T=300.0 K; NS=16
f2 (ppm)
f1 (ppm)

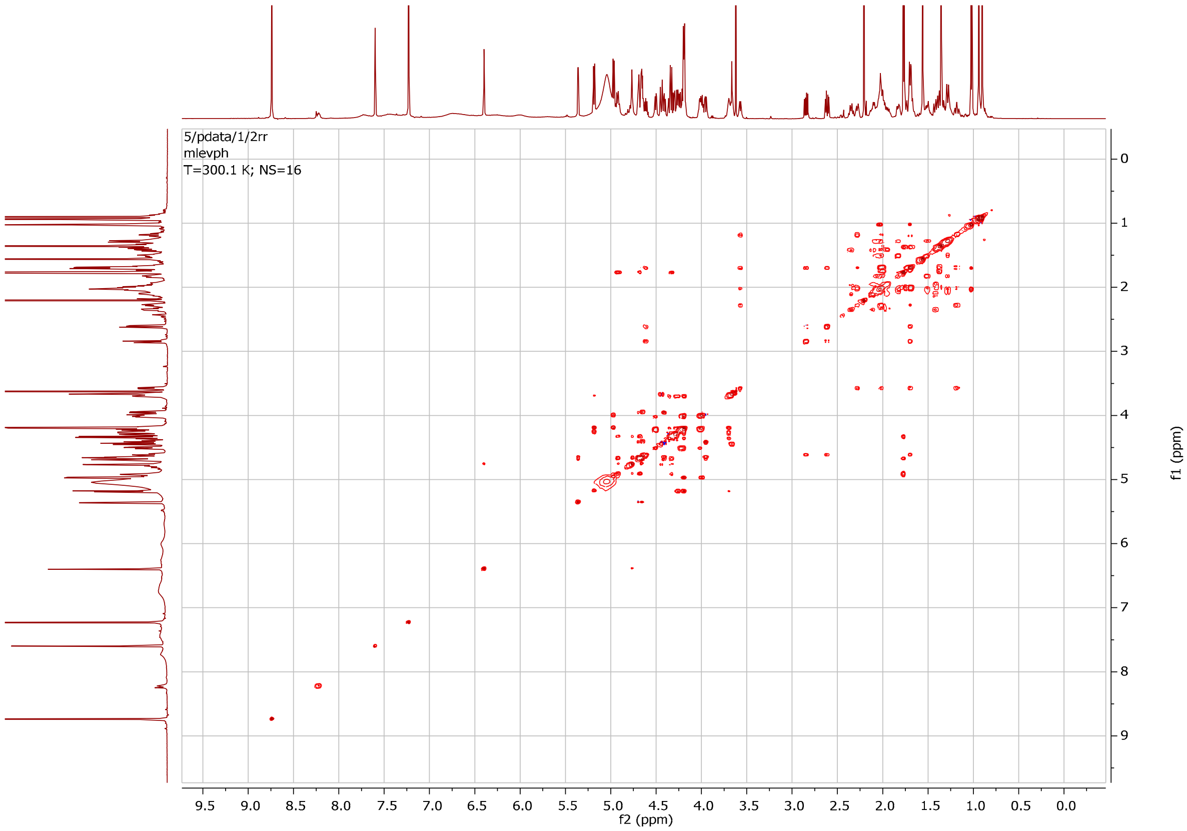
5/pdata/1/2rr
mlevph
T=300.1 K; NS=16
f2 (ppm)
f1 (ppm)

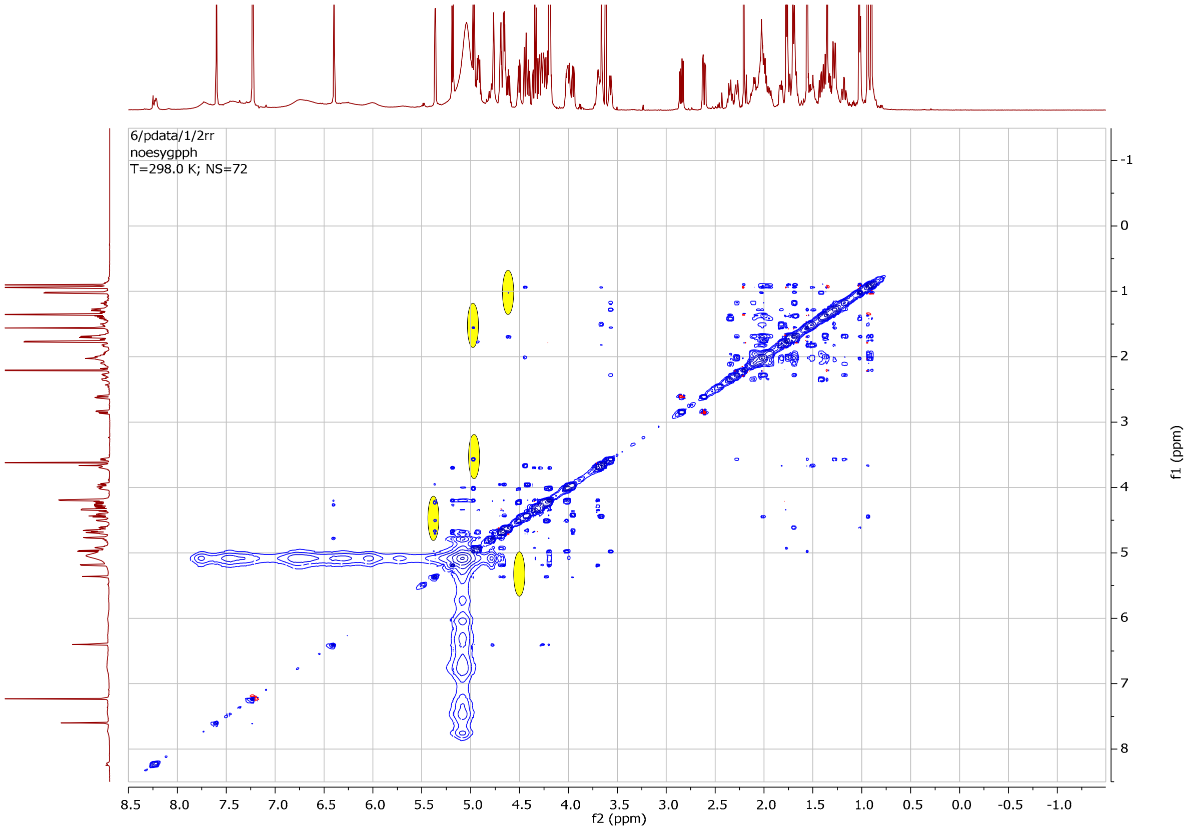
6/pdata/1/2rr
noesygpph
T=298.0 K; NS=72
f2 (ppm)
f1 (ppm)
